# Crystal structures and Hirshfeld surface analyses of 6,8-dimeth­oxy-3-methyl-1*H*-isochromen-1-one and 5-bromo-6,8-dimeth­oxy-3-methyl-1*H*-isochromen-1-one chloro­form monosolvate

**DOI:** 10.1107/S2056989020007975

**Published:** 2020-06-19

**Authors:** Mustapha Tiouabi, Raphaël Tabacchi, Helen Stoeckli-Evans

**Affiliations:** a Institute of Chemistry, University of Neuchâtel, Av. de Bellevax 51, CH-2000 Neuchâtel, Switzerland; b Institute of Physics, University of Neuchâtel, rue Emile-Argand 11, CH-2000 Neuchâtel, Switzerland

**Keywords:** crystal structure, isocoumarin, isochromen-1-one, hydrogen bonding, C—H⋯π inter­actions, offset π-π– inter­actions, Hirshfeld surface analysis

## Abstract

In the mol­ecule of 6,8-dimeth­oxy-3-methyl-1*H*-isochromen-1-one, C_12_H_12_O_4_, (**I**), the two meth­oxy groups are directed *anti* with respect to each other. In the mol­ecule of the brom­inated derivative, 5-bromo-6,8-dimeth­oxy-3-methyl-1*H*-isochromen-1-one, that crystallized as a chloro­form monosolvate, C_12_H_11_BrO_4_·CHCl_3_, (**II·CHCl_3_**), the meth­oxy groups are directed *syn* to each other. In the crystal of **I**, mol­ecules are linked by bifurcated C—H⋯O hydrogen bonds, forming chains along the *c*-axis direction. The chains are linked by C—H⋯π inter­actions, forming a supra­molecular framework. In the crystal of **II·CHCl_3_**, mol­ecules are linked by C—H⋯O hydrogen bonds forming 2_1_ helices parallel to the *b*-axis direction. The chloro­form solvate mol­ecules are linked to the helices by C—H⋯O(carbon­yl) hydrogen bonds. The helices stack up the *c*-axis direction and are linked by offset π–π inter­actions [inter­centroid distance = 3.517 (3) Å], forming layers parallel to the (100) plane. Compound **II·CHCl_3_** was refined as a two-component twin. Two chlorine atoms of the chloro­form solvate are disordered over two positions and were refined with a fixed occupancy ratio of 0.5:0.5.

## Abstract 2

Bromination of 6,8-dimeth­oxy-3-methyl-1*H*-isochromen-1-one resulted in the formation of the 5-bromo derivative, 5-bromo-6,8-dimeth­oxy-3-methyl-1*H*-isochromen-1-one. The two mol­ecules differ essentially in the orientation of the meth­oxy group on position 6 of the isocoumarin ring system.

## Chemical context   

Compound **I** is the protected form of the isocoumarin 6,8-dihy­droxy-3-methyl-1*H*-isochromen-1-one (**L**), which is a phytotoxin produced by the *Ceratocystis fimbriata* species *coffea* and *platani* (Gremaud & Tabacchi, 1994 ▸; Bürki *et al.*, 2003 ▸). These fungi are pathogenic agents responsible for infections of coffee, plane and elm trees (Michel, 2001 ▸). Compound **L** has also been isolated from the organic extracts of the fungus *Ceratocystis minor* (Hemingway *et al.*, 1977 ▸). The crystal structure of **L** has been reported for a sample obtained from the fermented culture of the endophytic marine fungus *Cephalosporium sp.* (Shao *et al.*, 2009 ▸). Herein, we report on the crystal structures and Hirshfeld surface analyses of the 6,8-dimeth­oxy derivative of **L**, *viz*. 6,8-dimeth­oxy-3-methyl-1*H*-isochromen-1-one (**I**) and compound **II**, 5-bromo-6,8-dimeth­oxy-3-methyl-1*H*-isochromen-1-one, the brominated derivative of **I**. The syntheses of compounds **I** and **II** were undertaken during the syntheses of derivatives of natural isocoumarins, metabolites of the pathogenic fungus *Ceratocystis fimbriata* sp. (Tiouabi, 2005 ▸).
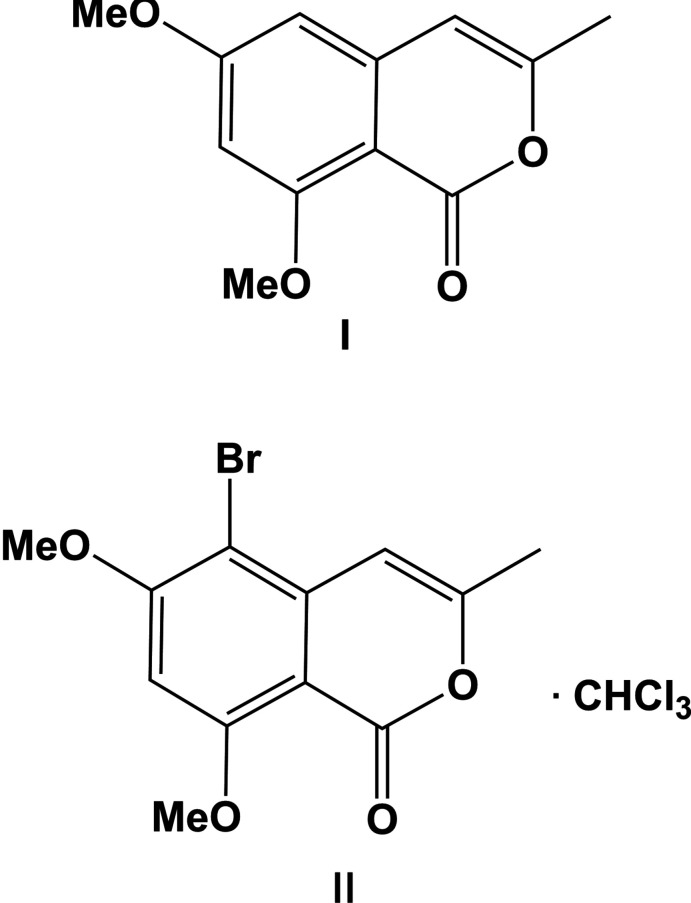


## Structural commentary   

**Figure 1:**
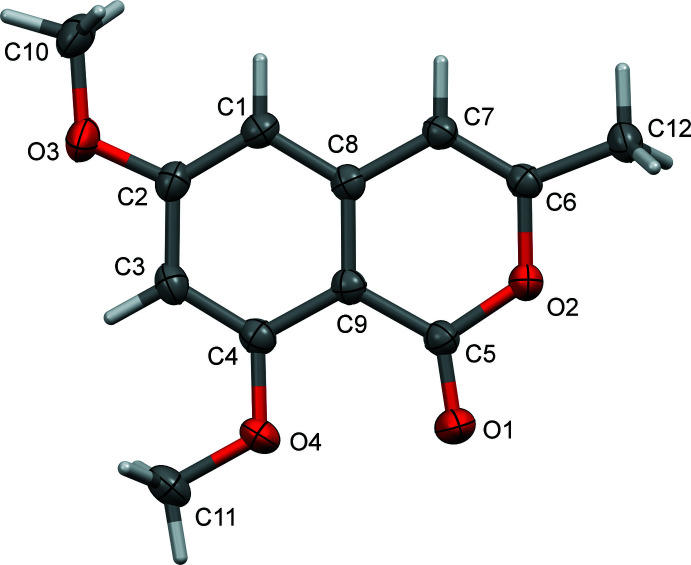
The mol­ecular structure of compound **I**, with atom labelling. Displacement ellipsoids are drawn at the 50% probability level.

**Figure 3:**
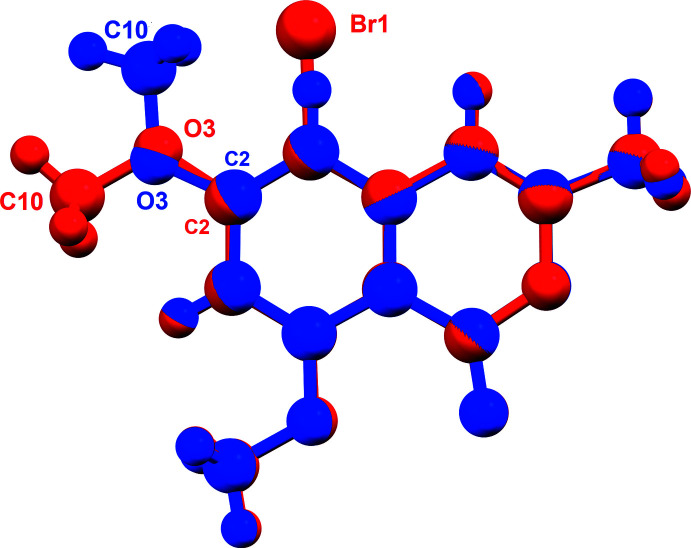
The structural overlap of compounds **I** (blue) and **II** (red); r.m.s. deviation = 0.0107 Å (*Mercury*; Macrae *et al.*, 2020 ▸).

The mol­ecular structures of compounds **I** and **II** are illustrated in Figs. 1 ▸ and 2 ▸, respectively. Compound **II** crystallized as a chloro­form monosolvate. Both isocoumarin mol­ecules are essentially planar with an r.m.s. deviation of 0.02 Å for **I** and 0.016 Å for **II** (H atoms not included). The maximum deviation from their mean planes is 0.047 (1) Å for atom O2 in **I**, and 0.035 (8) Å for atom C10 in **II**. The two mol­ecules differ essentially in the orientation of the meth­oxy group on atom C2. In **I** it is *anti* with respect to that on atom C4, while in **II**, owing to the steric hindrance of the Br atom, it has been rotated by 180° about the C2—O3 bond and is positioned *syn* with respect to the meth­oxy group on atom C4 (Fig. 3 ▸).

## Supra­molecular features   

**Figure 4:**
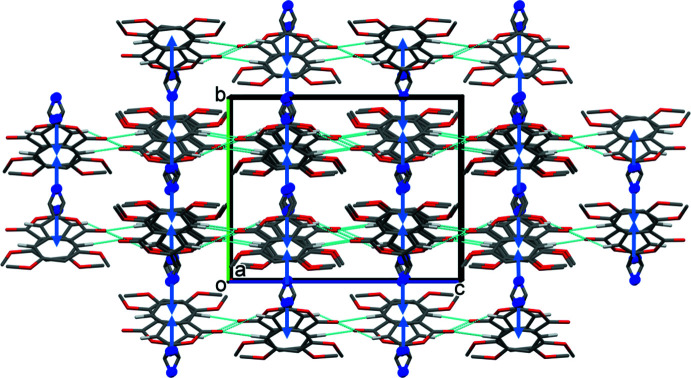
A view along the *a* axis of the crystal packing of compound **I**. The hydrogen bonds (Table 1 ▸) are shown as dashed lines and the C—H⋯π inter­actions as blue arrows. For clarity, only the H atoms (grey sticks and blue balls) involved in these inter­actions have been included.

The crystal packing of compound **I** is illustrated in Fig. 4 ▸. Mol­ecules are linked by bifurcated C—H⋯O hydrogen bonds, C1—H1⋯O1^i^ and C7—H7⋯O1^i^, forming chains propagating along the *c*-axis direction (Table 1 ▸). The chains are linked by C—H⋯π inter­actions (C12—H12*A*⋯*Cg* ^ii^ and C12—H12*B*⋯*Cg* ^iii^, where *Cg* is the centroid of the C1–C4/C8/C9 benzene ring), forming a supra­molecular framework (Table 1 ▸ and Fig. 4 ▸).

**Figure 5:**
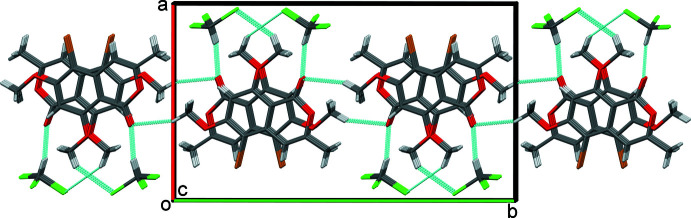
A view along the *c* axis of the crystal packing of compound **II·CHCl_3_**. The hydrogen bonds (Table 2 ▸) are shown as dashed lines.

In the crystal of **II·CHCl_3_**, mol­ecules are linked by C—H⋯O hydrogen bonds, C10—H10*C*⋯O1^i^, forming 2_1_ helices lying parallel to the *b*-axis direction (Table 2 ▸ and Fig. 5 ▸). The chloro­form solvate mol­ecules are linked to the helices by C—H⋯Cl and C—H⋯O hydrogen bonds, C11—H11*C*⋯Cl3*A* and C20—H20⋯O1 (Table 2 ▸). The helices stack up the *c*-axis direction and are linked by offset π–π inter­actions: *Cg*⋯*Cg* ^ii^ = 3.517 (3) Å, where *Cg* is the centroid of the C1–C4/C8/C9 benzene ring; α = 0.7 (3)°, β = 19.2°, γ = 19.8°, inter­planar distances are 3.359 (2) and 3.373 (2) Å, offset = 1.173 Å, symmetry code: (ii) *x*, −*y* + 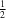, *z* − 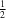. These latter inter­actions result in the formation of layers lying parallel to the *bc* plane (Fig. 5 ▸). There are no inter-layer contacts present.

## Hirshfeld surfaces and fingerprint plots for I and II·CHCl_3_   

The Hirshfeld surface analysis (Spackman & Jayatilaka, 2009 ▸) and the calculation of the associated two-dimensional fingerprint plots (McKinnon *et al.*, 2007 ▸) were performed with *CrystalExplorer17.5* (Turner *et al.*, 2017 ▸), following the protocol of Tiekink and collaborators (Tan *et al.*, 2019 ▸). The Hirshfeld surface is colour-mapped with the normalized contact distance, *d*_norm_, from red (distances shorter than the sum of the van der Waals radii) through white to blue (distances longer than the sum of the van der Waals radii).

**Figure 6:**
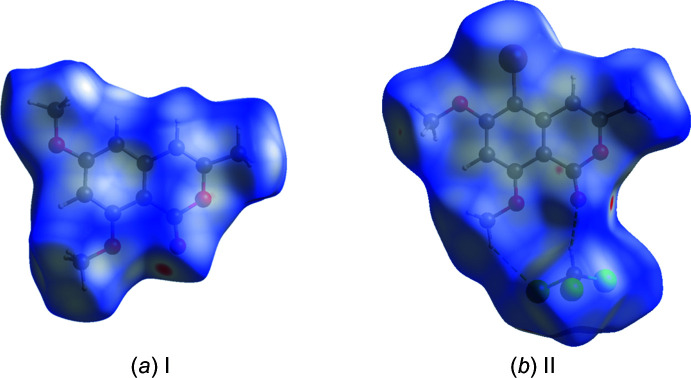
(*a*) The Hirshfeld surface of compound **I**, mapped over *d*_norm_, in the colour range −0.1596 to 1.1682 a.u., (*b*) the Hirshfeld surface of compound **II·CHCl_3_**, mapped over *d*_norm_, in the colour range −0.0734 to 1.3731 a.u.. The dashed lines indicate the hydrogen bonds linking the two units (see Table 2 ▸).

A summary of the short inter­atomic contacts in **I** and **II·CHCl_3_** is given in Table 3 ▸. The Hirshfeld surfaces of **I** and **II** mapped over *d*_norm_, are shown in Fig. 6 ▸ *a* and *b*, respectively. The faint red spots indicate that short contacts are significant in the crystal packing of both compounds.

**Figure 7:**
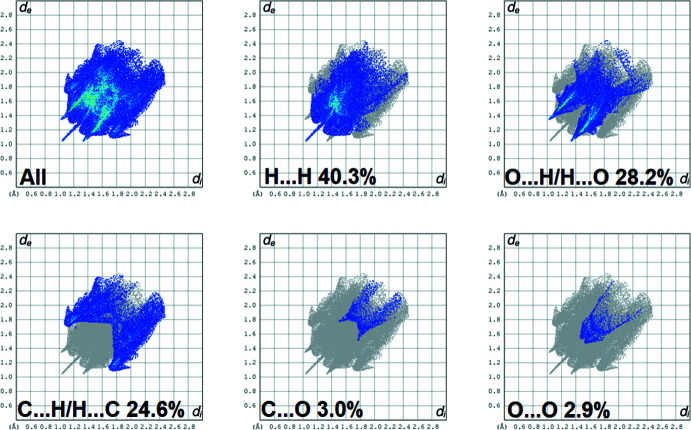
The full two-dimensional fingerprint plot for compound **I**, and fingerprint plots delineated into H⋯H (40.3%), O⋯H/H⋯O (28.2%), C⋯H/H⋯C (24.6%), C⋯O (3.0%), and O⋯O (2.9%) contacts.

The full two-dimensional fingerprint plot for **I** and fingerprint plots delineated into H⋯H (40.3%), O⋯H/H⋯O (28.2%), C⋯H/H⋯C (24.6%), C⋯O (3.0%) and O⋯O (2.9%) contacts, are shown in Fig. 7 ▸. The C⋯C contacts contribute only 1.0%.

**Figure 8:**
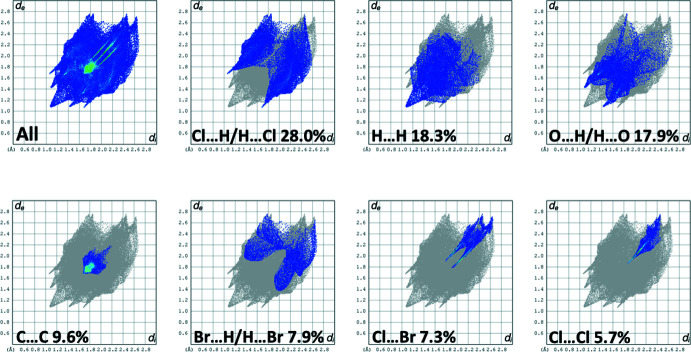
The full two-dimensional fingerprint plot for compound **II·CHCl_3_**, and fingerprint plots delineated into Cl⋯H/H⋯Cl (28.0%), H⋯H (18.3%), O⋯H/H⋯O (17.9%), C⋯C (9.6%), Br⋯H/H⋯Br (7.9%), Cl⋯Br (7.3%) and Cl⋯Cl (5.7%),contacts.

The full two-dimensional fingerprint plot for compound **II·CHCl_3_**, and fingerprint plots delineated into Cl⋯H/H⋯Cl (28.0%), H⋯H (18.3%), O⋯H/H⋯O (17.9%), C⋯C (9.6%), Br⋯H/H⋯Br (7.9%), Cl⋯Br (7.3%) and Cl⋯Cl (5.7%) contacts are shown in Fig. 8 ▸. The C⋯O contacts contribute 2.2% but the C⋯H/H⋯C contacts contribute only 1.2% compared to 24.6% in **I**.

The H⋯H contacts in **II·CHCl_3_** (18.3%) are considerably reduced compared to those in **I (**H⋯H at 40.3%), while the Cl⋯H/H⋯Cl (28.0%) and O⋯H/H⋯O (17.9%) contacts dominate the inter­atomic contacts and combined are stronger that those in **I** (O⋯H/H⋯O at 28.2%).

## Database survey   

A search of the Cambridge Structural Database (CSD, Version 5.41, last update March 2020; Groom *et al.*, 2016 ▸) for the 1*H*-isochromen-1-one skeleton gave 217 hits. Only one compound contains 6,8-dimeth­oxy substituents, *viz*. 5,6,8-trimeth­oxy-3,4,7-tri­methyl­isocoumarin (CSD refcode JICLOW; Botha *et al.*, 1991 ▸). A search for the 3-methyl-1*H*-isochromen-1-one substructure gave 16 hits. Apart from the structure of 3-methyl-1*H*-isochromen-1-one itself (GECYUK; Liu *et al.*, 2012 ▸), the most important structure is that for 6,8-dihy­droxy-3-methyl-1*H*-isochromen-1-one (MOSLOW; Shao *et al.*, 2009 ▸), *viz*. compound **L** described above (see §1. *Chemical context*).

## Synthesis and crystallization   

**Figure 9:**
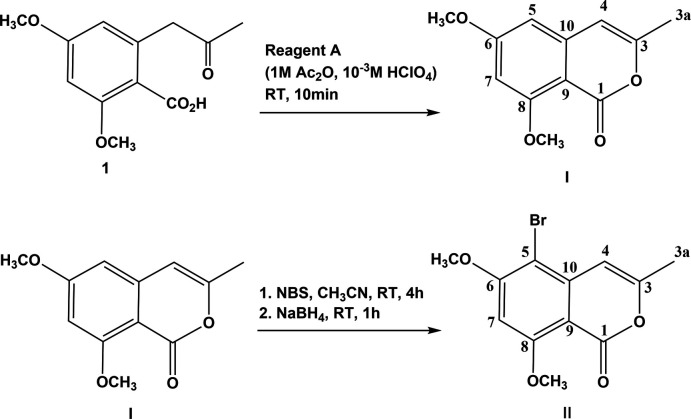
Reaction schemes for the syntheses of compounds **I** and **II**, with atom-labelling schemes in relation to the NMR spectra (see §6. *Synthesis and crystallization*).

The syntheses of compounds **I** and **II** are illustrated in Fig. 9 ▸, together with the atom labelling in relation to the NMR spectra. The syntheses of the keto-acid, 1,2,4-dimeth­oxy-6-(2-oxoprop­yl)benzoic acid (**1**), together with compounds **I** and **II** were undertaken during the syntheses of derivatives of natural isocoumarins, metabolites of the pathogenic fungus *Ceratocystis fimbriata* sp. (Tiouabi, 2005 ▸).

**Preparation of** ***Reagent A*** **(1** ***M*** **Ac_2_O; 10^−3^** ***M*** **HClO_4_)**, was carried out according to the protocol of Edwards & Rao (Edwards & Rao, 1966 ▸). 0.0501 ml of HClO_4_ at 70% (0.575 mmol) were dissolved in 50 ml of AcOEt. 30 ml of this solution were added to a solution of 14.4 ml of Ac_2_O (0.153 mol) in 105.6 ml of AcOEt to give 150 ml of *Reagent A*.

**Synthesis of 6,8-dimeth­oxy-3-methyl-1** ***H*** **-isochromen-1-one (I):** In a 250 ml flask equipped with a magnetic stirrer and under an atmosphere of argon, the keto-acid (**1**) was dissolved in 150 ml of *Reagent A.* The mixture was stirred vigorously for 10–15 min, then washed with an aqueous solution of saturated NaHCO_3_. The organic phase was dried over anhydrous Na_2_SO_4_, then filtered and the filtrate concentrated using rotary evaporation. The brown solid obtained was purified by chromatography on a silica column using as eluent CH_2_Cl_2_/AcOEt (15/1, *v*/*v*). On evaporation of the eluent 1.20 g of compound **I** (yield 95%) were obtained as colourless block-like crystals.

***Analytical data for I:** R*_f_ (CH_2_Cl_2_/MeOH: 20/0.5; UV) 0.735. ^1^H NMR (400 MHz, CDCl_3_, 298 K): 2.16 [*d*, 4*J*(3a-4) = 1, 3H, CH_3_], 3.84 (*s*, 3H, OCH_3_), 3.90 (*s*, 3H, OCH_3_), 6.03 [*q*, 4*J*(4-3a) = 1.0, 1H, H-4], 6.24 (*d*, *J*_m_ = 2.3, 1H, ArH-5), 6.36 (*d*, *J*_m_ = 2.3, 1H, ArH-7). ^13^C NMR (100 Hz, CDCl_3_, 298 K, HETCOR–SR/LR): 19.82 C(3a), 55.97 C(OCH_3_, 56.59 C(OCH_3_), 98.46 C(7), 99.80 C(5), 103.04 C(9), 104.08 C(4), 142.80 C(10), 155.79 C(3), 159.97 C(1), 163.56 C(8), 165.75 C(6). MS [ESI(+)]: ms 243.1 [*M* + Na]^+^; ms 221.3 [*M* + H]^+^. HR–MS (ESI(+)): ms 243.06256 [*M* + Na]^+^. IR (KBr disk, cm^−1^): 1713 *vs*, 1667 *m*, 1599 *vs*, 1168 *m*, 969 *m*.

**Synthesis of 5-bromo-6,8-dimeth­oxy-3-methyl-1** ***H*** **-isochro­men-1-one (II):** In a 25 ml flask equipped with a magnetic stirrer and under an atmosphere of argon, NBS (*N*-bromosuccinimide) (28 mg, 0.158 mmol) was added under stirring to a solution of compound **I** (0.136 mmol) dissolved in CH_3_CN (1.5 ml). The reaction mixture was stirred for 2 h at room temperature. On completion of the reaction, followed by thin-layer chromatography using CH_2_Cl_2_/AcOEt (15/2, *v*/*v*) as eluent, NaBH_4_ (5.2 mg, 0.136 mmol) was added, resulting in the transformation of the yellow solution into a white suspension. After 1 h the reaction mixture was diluted using water and then extracted five times using AcOEt. The organic phases were combined, dried over anhydrous Na_2_SO_4_, then filtered and the filtrate concentrated using rotary evaporation. The white solid obtained was purified by chromatography on a silica column using CH_2_Cl_2_/AcOEt (20/1, *v*/*v*) as eluent. On evaporation of the eluent, 30 mg of compound **II** (yield 74%) were obtained as colourless rod-like crystals.

***Analytical data for II***: *R*_f_ (CH_2_Cl_2_/AcOEt: 15/2, UV) 0.26. ^1^H NMR (400 MHz, CDCl_3_, 298K): 2.26 [*d*, 4*J*(3a-4) = 0.8, 3H, CH_3_], 4.02 (*s*, 6H, 2 × OCH3), 6.45 (*s*, 1H, ArH-7), 6.58 [*q*, 4*J*(4-3a) = 0.8, 1H, H-4]. ^13^C NMR (100 Hz, CDCl_3_, 298K, HETCOR–SR): 20.31 C(3a), 56.80 C(OCH_3_), 56.90 C(OCH_3_), 94.67 C(7), 98.47 C(5), 102.73 C(4), 103.56 C(9), 140.41 C(10), 156.86 C(3), 159.28 C(1), 161.67 C(8), 163.37 C(6). MS[ESI(+)]: ms 299.1 [*M*(79Br) + H]^+^, ms 301.1 [*M*(81Br) + H]^+^. HR–MS [ESI(+)]: ms 320.97315 [*M*(79Br) + Na]^+^, ms 322.97144 [*M*(81Br) + Na]^+^. IR (KBr disk, cm^−1^): 1724 *vs*, 1667 *s*, 1580 *vs*, 1215 *vs*, 1038 *m*.

## Refinement   

Crystal data, data collection and structure refinement details are summarized in Table 4 ▸. For both **I** and **II** the C-bound H atoms were included in calculated positions and treated as riding on their parent C atom: C—H = 0.95–1.00 Å with *U*_iso_(H) = 1.5*U*_eq_(C-meth­yl) and 1.2*U*_eq_(C) for other H atoms.

Compound **II·CHCl_3_** was refined as a two-component twin with a 180° rotation about axis c*. Details are given in the archived CIF. The final refined BASF factor is 0.2590 (19). Two of the chloro­form solvate chlorine atoms (Cl2 and Cl3) are disordered over two positions and were refined with a fixed occupancy ratio (Cl2*A*:Cl2*B* and Cl3*A*:Cl3*B*) of 0.5:0.5.

## Supplementary Material

Crystal structure: contains datablock(s) I, II, Global. DOI: 10.1107/S2056989020007975/dj2010sup1.cif

Structure factors: contains datablock(s) I. DOI: 10.1107/S2056989020007975/dj2010Isup2.hkl

Structure factors: contains datablock(s) II. DOI: 10.1107/S2056989020007975/dj2010IIsup3.hkl

CCDC references: 2009829, 2009830

Additional supporting information:  crystallographic information; 3D view; checkCIF report

## Figures and Tables

**Figure 2:**
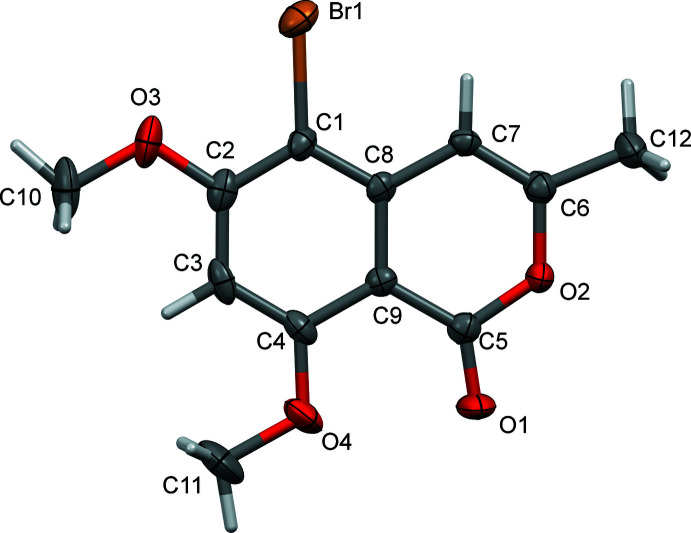
The mol­ecular structure of compound **II**, with atom labelling. Displacement ellipsoids are drawn at the 50% probability level. For clarity, the chloro­form solvate mol­ecule has been omitted.

**Table 1:** Hydrogen-bond geometry (Å, °) for **I** [Chem scheme1] *Cg* is the centroid of the C1–C4/C8/C9 benzene ring.

*D*—H⋯*A*	*D*—H	H⋯*A*	*D*⋯*A*	*D*—H⋯*A*
C1—H1⋯O1^i^	0.95	2.55	3.366 (2)	144
C7—H7⋯O1^i^	0.95	2.50	3.3269 (19)	146
C12—H12*A*⋯*Cg* ^ii^	0.98	2.67	3.4902 (18)	141
C12—H12*B*⋯*Cg* ^iii^	0.98	2.88	3.5456 (18)	126

Symmetry codes: (i) 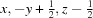; (ii) 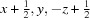; (iii) 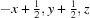.

**Table 2:** Hydrogen-bond geometry (Å, °) for **II·CHCl_3_** [Chem scheme1]

*D*—H⋯*A*	*D*—H	H⋯*A*	*D*⋯*A*	*D*—H⋯*A*
C10—H10*C*⋯O1^i^	0.98	2.59	3.511 (6)	156
C11—H11*C*⋯Cl3*A*	0.98	2.79	3.629 (9)	144
C20—H20⋯O1	1.00	2.15	3.126 (6)	164

Symmetry code: (i) 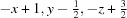.

**Table 3:** Short inter­atomic contacts^*a*^ (Å) for **I** and **II·CHCl_3_**

Atom1	Atom2	Length	Length − VdW	Symm. code Atom 2
**I**				
H7	O1	2.497	−0.223	*x*, 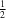 − *y*, − 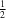 + *z*
H1	O1	2.551	−0.169	*x*, 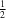 − *y*, − 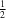 + *z*
H10*A*	H10*A*	2.281	−0.119	−*x*, −*y*, −*z*
H11*C*	O2	2.683	−0.037	− 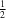 + *x*, 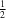 − *y*, 1 − *z*
H11*B*	O1	2.691	−0.029	− 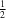 + *x*, 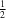 − *y*, 1 − *z*
O3	O2	3.023	−0.017	−*x*, − 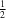 + *y*, 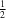 − *z*
H10*B*	C11	2.903	0.003	*x*, 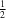 − *y*, − 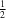 + *z*
C10	H12*C*	2.911	0.011	− 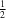 + *x*, 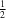 − *y*, −*z*
C11	C11	3.411	0.011	−*x*, −*y*, 1 − *z*
O4	H11*A*	2.735	0.015	−*x*, −*y*, 1 − *z*
C8	H12*B*	2.915	0.015	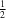 − *x*, − 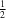 + *y*, *z*
C8	H12*A*	2.920	0.020	− 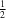 + *x*, *y*, 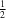 − *z*
C1	H12*A*	2.938	0.038	− 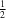 + *x*, *y*, 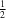 − *z*
H1	O4	2.763	0.043	*x*, 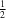 − *y*, − 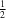 + *z*
C11	H11*A*	2.978	0.078	−*x*, −*y*, 1 − *z*
C9	H12*B*	2.984	0.084	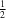 − *x*, − 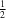 + *y*, *z*
O3	C6	3.310	0.090	−*x*, − 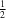 + *y*, 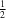 − *z*
H10*C*	C11	2.997	0.097	− 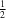 − *x*, −*y*, − 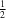 + *z*
H10*B*	O1	2.819	0.099	− 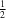 + *x*, *y*, 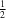 − *z*
				
**II·CHCl_3_**				
O1	H20	2.154	−0.566	*x*, *y*, *z*
H11*C*	Cl3*A*	2.793	−0.157	*x*, *y*, *z*
H10*C*	O1	2.595	−0.125	1 − *x*, − 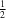 + *y*, 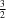 − *z*
H10*A*	H10*A*	2.291	−0.109	1 − *x*, −*y*, 2 − *z*
O1	C20	3.126	−0.094	*x*, *y*, *z*
H7	Cl3*A*	2.871	−0.079	−1 + *x*, *y*, *z*
C3	C5	3.375	−0.025	*x*, 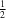 − *y*, − 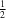 + *z*
C10	O2	3.196	−0.024	1 − *x*, − 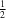 + *y*, 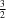 − *z*
C1	C8	3.399	−0.001	*x*, 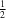 − *y*, − 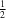 + *z*
H11*A*	Cl1	2.961	0.011	*x*, 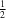 − *y*, − 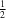 + *z*
C4	C4	3.432	0.032	*x*, 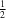 − *y*, − 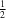 + *z*
H10*C*	O2	2.754	0.034	1 − *x*, − 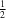 + *y*, 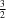 − *z*
Br1	C7	3.591	0.041	*x*, 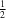 − *y*, − 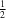 + *z*
C1	C7	3.463	0.063	*x*, 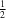 − *y*, − 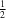 + *z*
C8	C8	3.485	0.085	*x*, 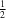 − *y*, − 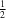 + *z*
C11	Cl1	3.538	0.088	*x*, 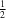 − *y*, − 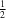 + *z*
C9	C4	3.495	0.095	*x*, 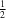 − *y*, − 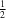 + *z*

(*a*) Calculated using *Mercury* (Macrae *et al.*, 2020 ▸).

**Table 4:** Experimental details

	**I**	**II·CHCl_3_**
Crystal data		
Chemical formula	C_12_H_12_O_4_	C_12_H_11_BrO_4_·CHCl_3_
*M*_r_	220.22	418.49
Crystal system, space group	Orthorhombic, *P* *b* *c* *a*	Monoclinic, *P*2_1_/*c*
Temperature (K)	173	173
*a*, *b*, *c* (Å)	12.7875 (9), 11.3732 (12), 14.3637 (12)	11.7655 (9), 20.4640 (17), 6.7332 (5)
α, β, γ (°)	90, 90, 90	90, 90.161 (9), 90
*V* (Å^3^)	2089.0 (3)	1621.1 (2)
*Z*	8	4
Radiation type	Mo *K*α	Mo *K*α
μ (mm^−1^)	0.11	3.04
Crystal size (mm)	0.36 × 0.28 × 0.26	0.30 × 0.11 × 0.10

Data collection		
Diffractometer	Stoe IPDS 2	Stoe IPDS 1
Absorption correction	Multi-scan (*MULABS*; Spek, 2020 ▸)	Multi-scan (*MULABS*; Spek, 2020 ▸)
*T*_min_, *T*_max_	0.903, 1.000	0.894, 1.000
No. of measured, independent and observed [*I* > 2σ(*I*)] reflections	24778, 2835, 1990	3131, 3131, 2066
*R*_int_	0.077	0.087
(sin θ/λ)_max_ (Å^−1^)	0.689	0.616

Refinement		
*R*[*F* ^2^ > 2σ(*F* ^2^)], *wR*(*F* ^2^), *S*	0.052, 0.116, 1.05	0.040, 0.100, 0.88
No. of reflections	2835	3131
No. of parameters	148	211
H-atom treatment	H-atom parameters constrained	H-atom parameters constrained
Δρ_max_, Δρ_min_ (e Å^−3^)	0.22, −0.18	0.57, −0.40

Computer programs: *X-AREA* and *X-RED32* (Stoe & Cie, 2005 ▸), *EXPOSE*, *CELL* and *INTEGRATE* in *IPDS-I* (Stoe & Cie, 2004 ▸), *SHELXS97* (Sheldrick, 2008 ▸), *SHELXL2018/3* (Sheldrick, 2015 ▸), *PLATON* (Spek, 2020 ▸), *Mercury* (Macrae *et al.*, 2020 ▸) and *publCIF* (Westrip, 2010 ▸).

## supplementary crystallographic information

### 6,8-Dimethoxy-3-methyl-1H-isochromem-1-one (I) Crystal data

**Table:**

C_12_H_12_O_4_	*D*_x_ = 1.400 Mg m^−^^3^
*M**_r_* = 220.22	Mo *K*α radiation, λ = 0.71073 Å
Orthorhombic, *P**b**c**a*	Cell parameters from 10689 reflections
*a* = 12.7875 (9) Å	θ = 1.7–29.6°
*b* = 11.3732 (12) Å	µ = 0.11 mm^−^^1^
*c* = 14.3637 (12) Å	*T* = 173 K
*V* = 2089.0 (3) Å^3^	Block, colourless
*Z* = 8	0.36 × 0.28 × 0.26 mm
*F*(000) = 928	

### 6,8-Dimethoxy-3-methyl-1H-isochromem-1-one (I) Data collection

**Table:**

STOE IPDS 2 diffractometer	2835 independent reflections
Radiation source: fine-focus sealed tube	1990 reflections with *I* > 2σ(*I*)
Plane graphite monochromator	*R*_int_ = 0.077
φ + ω scans	θ_max_ = 29.3°, θ_min_ = 2.8°
Absorption correction: multi-scan (MULABS; Spek, 2020)	*h* = −17→16
*T*_min_ = 0.903, *T*_max_ = 1.000	*k* = −15→15
24778 measured reflections	*l* = −19→19

### 6,8-Dimethoxy-3-methyl-1H-isochromem-1-one (I) Refinement

**Table:**

Refinement on *F*^2^	Primary atom site location: structure-invariant direct methods
Least-squares matrix: full	Secondary atom site location: difference Fourier map
*R*[*F*^2^ > 2σ(*F*^2^)] = 0.052	Hydrogen site location: inferred from neighbouring sites
*wR*(*F*^2^) = 0.116	H-atom parameters constrained
*S* = 1.05	*w* = 1/[σ^2^(*F*_o_^2^) + (0.051*P*)^2^ + 0.3781*P*] where *P* = (*F*_o_^2^ + 2*F*_c_^2^)/3
2835 reflections	(Δ/σ)_max_ < 0.001
148 parameters	Δρ_max_ = 0.22 e Å^−^^3^
0 restraints	Δρ_min_ = −0.18 e Å^−^^3^

### 6,8-Dimethoxy-3-methyl-1H-isochromem-1-one (I) Special details

**Table:**

Geometry. All esds (except the esd in the dihedral angle between two l.s. planes) are estimated using the full covariance matrix. The cell esds are taken into account individually in the estimation of esds in distances, angles and torsion angles; correlations between esds in cell parameters are only used when they are defined by crystal symmetry. An approximate (isotropic) treatment of cell esds is used for estimating esds involving l.s. planes.

### 6,8-Dimethoxy-3-methyl-1H-isochromem-1-one (I) Fractional atomic coordinates and isotropic or equivalent isotropic displacement parameters (Å^2^)

**Table:**

	*x*	*y*	*z*	*U*_iso_*/*U*_eq_	
O1	0.15786 (9)	0.27567 (12)	0.46504 (8)	0.0395 (3)	
O2	0.23972 (8)	0.33408 (10)	0.33972 (7)	0.0268 (3)	
O3	−0.14208 (10)	0.07550 (12)	0.14615 (9)	0.0404 (3)	
O4	−0.01901 (9)	0.15403 (11)	0.44753 (8)	0.0314 (3)	
C1	0.01713 (12)	0.18950 (14)	0.16114 (11)	0.0266 (3)	
H1	0.024720	0.199167	0.095797	0.032*	
C2	−0.06630 (12)	0.12755 (14)	0.19739 (11)	0.0282 (3)	
C3	−0.07906 (12)	0.11400 (14)	0.29319 (12)	0.0292 (3)	
H3	−0.136951	0.070662	0.316450	0.035*	
C4	−0.00875 (12)	0.16271 (14)	0.35416 (11)	0.0258 (3)	
C5	0.15644 (12)	0.27686 (14)	0.38113 (11)	0.0263 (3)	
C6	0.24844 (12)	0.34840 (13)	0.24518 (10)	0.0248 (3)	
C7	0.17841 (12)	0.30230 (14)	0.18753 (10)	0.0248 (3)	
H7	0.186695	0.312124	0.122268	0.030*	
C8	0.09018 (11)	0.23769 (13)	0.22263 (10)	0.0233 (3)	
C9	0.07889 (11)	0.22557 (13)	0.31981 (10)	0.0238 (3)	
C10	−0.13653 (15)	0.08442 (17)	0.04763 (13)	0.0387 (4)	
H10A	−0.068771	0.054680	0.026132	0.058*	
H10B	−0.144175	0.166918	0.029140	0.058*	
H10C	−0.192803	0.037821	0.019638	0.058*	
C11	−0.10579 (14)	0.08855 (17)	0.48242 (13)	0.0372 (4)	
H11A	−0.101267	0.007171	0.460350	0.056*	
H11B	−0.171053	0.123974	0.460230	0.056*	
H11C	−0.104631	0.089534	0.550635	0.056*	
C12	0.34151 (12)	0.42031 (15)	0.22198 (12)	0.0317 (4)	
H12A	0.404473	0.381766	0.246095	0.048*	
H12B	0.334533	0.498334	0.250262	0.048*	
H12C	0.347075	0.428395	0.154231	0.048*	

### 6,8-Dimethoxy-3-methyl-1H-isochromem-1-one (I) Atomic displacement parameters (Å^2^)

**Table:**

	*U*^11^	*U*^22^	*U*^33^	*U*^12^	*U*^13^	*U*^23^
O1	0.0350 (6)	0.0600 (9)	0.0237 (6)	−0.0096 (6)	−0.0017 (5)	0.0011 (5)
O2	0.0232 (5)	0.0312 (6)	0.0260 (5)	−0.0034 (5)	−0.0004 (4)	−0.0008 (4)
O3	0.0388 (7)	0.0462 (8)	0.0363 (7)	−0.0187 (6)	−0.0090 (5)	0.0013 (6)
O4	0.0312 (6)	0.0374 (7)	0.0257 (6)	−0.0052 (5)	0.0067 (5)	0.0027 (5)
C1	0.0281 (7)	0.0279 (8)	0.0238 (7)	0.0008 (6)	−0.0029 (6)	−0.0004 (6)
C2	0.0268 (7)	0.0268 (8)	0.0310 (8)	−0.0025 (6)	−0.0053 (6)	−0.0013 (6)
C3	0.0249 (7)	0.0276 (8)	0.0352 (9)	−0.0042 (6)	0.0019 (6)	0.0040 (7)
C4	0.0248 (7)	0.0259 (8)	0.0266 (7)	0.0021 (6)	0.0022 (6)	0.0012 (6)
C5	0.0233 (7)	0.0312 (8)	0.0244 (7)	0.0007 (6)	0.0011 (6)	0.0005 (6)
C6	0.0229 (7)	0.0258 (7)	0.0258 (7)	0.0014 (6)	0.0025 (6)	0.0006 (6)
C7	0.0235 (7)	0.0273 (7)	0.0235 (7)	0.0009 (6)	0.0011 (6)	0.0013 (6)
C8	0.0216 (6)	0.0223 (7)	0.0260 (7)	0.0026 (6)	0.0004 (6)	0.0003 (6)
C9	0.0222 (7)	0.0244 (7)	0.0249 (7)	0.0021 (6)	0.0011 (6)	−0.0006 (6)
C10	0.0384 (9)	0.0419 (11)	0.0358 (9)	−0.0055 (8)	−0.0114 (8)	−0.0041 (8)
C11	0.0348 (9)	0.0403 (10)	0.0365 (9)	−0.0037 (8)	0.0118 (7)	0.0048 (8)
C12	0.0258 (7)	0.0349 (9)	0.0343 (8)	−0.0060 (7)	0.0006 (7)	0.0024 (7)

### 6,8-Dimethoxy-3-methyl-1H-isochromem-1-one (I) Geometric parameters (Å, º)

**Table:**

O1—C5	1.2056 (19)	C6—C7	1.328 (2)
O2—C6	1.3722 (18)	C6—C12	1.482 (2)
O2—C5	1.3825 (18)	C7—C8	1.438 (2)
O3—C2	1.3532 (19)	C7—H7	0.9500
O3—C10	1.421 (2)	C8—C9	1.410 (2)
O4—C4	1.3511 (19)	C10—H10A	0.9800
O4—C11	1.427 (2)	C10—H10B	0.9800
C1—C2	1.380 (2)	C10—H10C	0.9800
C1—C8	1.397 (2)	C11—H11A	0.9800
C1—H1	0.9500	C11—H11B	0.9800
C2—C3	1.394 (2)	C11—H11C	0.9800
C3—C4	1.372 (2)	C12—H12A	0.9800
C3—H3	0.9500	C12—H12B	0.9800
C4—C9	1.418 (2)	C12—H12C	0.9800
C5—C9	1.449 (2)		
			
C6—O2—C5	122.97 (12)	C1—C8—C9	121.25 (14)
C2—O3—C10	118.34 (14)	C1—C8—C7	120.23 (14)
C4—O4—C11	117.52 (13)	C9—C8—C7	118.52 (14)
C2—C1—C8	118.58 (14)	C8—C9—C4	118.34 (14)
C2—C1—H1	120.7	C8—C9—C5	119.49 (14)
C8—C1—H1	120.7	C4—C9—C5	122.18 (14)
O3—C2—C1	124.86 (15)	O3—C10—H10A	109.5
O3—C2—C3	113.86 (14)	O3—C10—H10B	109.5
C1—C2—C3	121.28 (14)	H10A—C10—H10B	109.5
C4—C3—C2	120.57 (14)	O3—C10—H10C	109.5
C4—C3—H3	119.7	H10A—C10—H10C	109.5
C2—C3—H3	119.7	H10B—C10—H10C	109.5
O4—C4—C3	122.72 (14)	O4—C11—H11A	109.5
O4—C4—C9	117.32 (14)	O4—C11—H11B	109.5
C3—C4—C9	119.97 (14)	H11A—C11—H11B	109.5
O1—C5—O2	115.07 (14)	O4—C11—H11C	109.5
O1—C5—C9	127.84 (15)	H11A—C11—H11C	109.5
O2—C5—C9	117.08 (13)	H11B—C11—H11C	109.5
C7—C6—O2	121.03 (14)	C6—C12—H12A	109.5
C7—C6—C12	128.27 (15)	C6—C12—H12B	109.5
O2—C6—C12	110.69 (13)	H12A—C12—H12B	109.5
C6—C7—C8	120.83 (14)	C6—C12—H12C	109.5
C6—C7—H7	119.6	H12A—C12—H12C	109.5
C8—C7—H7	119.6	H12B—C12—H12C	109.5
			
C10—O3—C2—C1	0.0 (2)	C2—C1—C8—C9	−1.0 (2)
C10—O3—C2—C3	179.81 (16)	C2—C1—C8—C7	179.69 (15)
C8—C1—C2—O3	−179.38 (16)	C6—C7—C8—C1	−179.92 (15)
C8—C1—C2—C3	0.8 (2)	C6—C7—C8—C9	0.8 (2)
O3—C2—C3—C4	−179.52 (15)	C1—C8—C9—C4	0.1 (2)
C1—C2—C3—C4	0.3 (3)	C7—C8—C9—C4	179.43 (14)
C11—O4—C4—C3	1.6 (2)	C1—C8—C9—C5	179.98 (14)
C11—O4—C4—C9	−178.59 (14)	C7—C8—C9—C5	−0.7 (2)
C2—C3—C4—O4	178.59 (15)	O4—C4—C9—C8	−178.83 (14)
C2—C3—C4—C9	−1.2 (2)	C3—C4—C9—C8	1.0 (2)
C6—O2—C5—O1	−177.08 (15)	O4—C4—C9—C5	1.3 (2)
C6—O2—C5—C9	3.2 (2)	C3—C4—C9—C5	−178.86 (15)
C5—O2—C6—C7	−3.2 (2)	O1—C5—C9—C8	179.13 (17)
C5—O2—C6—C12	175.88 (13)	O2—C5—C9—C8	−1.2 (2)
O2—C6—C7—C8	1.1 (2)	O1—C5—C9—C4	−1.0 (3)
C12—C6—C7—C8	−177.82 (15)	O2—C5—C9—C4	178.68 (14)

### 6,8-Dimethoxy-3-methyl-1H-isochromem-1-one (I) Hydrogen-bond geometry (Å, º)

*Cg* is the centroid of the C1–C4/C8/C9 benzene ring.

**Table:**

*D*—H···*A*	*D*—H	H···*A*	*D*···*A*	*D*—H···*A*
C1—H1···O1^i^	0.95	2.55	3.366 (2)	144
C7—H7···O1^i^	0.95	2.50	3.3269 (19)	146
C12—H12*A*···*Cg*^ii^	0.98	2.67	3.4902 (18)	141
C12—H12*B*···*Cg*^iii^	0.98	2.88	3.5456 (18)	126

Symmetry codes: (i) *x*, −*y*+1/2, *z*−1/2; (ii) *x*+1/2, *y*, −*z*+1/2; (iii) −*x*+1/2, *y*+1/2, *z*.

### 5-Bromo-6,8-dimethoxy-3-methyl-1H-isochromen-1-one chloroform monosolvate (II) Crystal data

**Table:**

C_12_H_11_BrO_4_·CHCl_3_	*F*(000) = 832
*M**_r_* = 418.49	*D*_x_ = 1.715 Mg m^−^^3^
Monoclinic, *P*2_1_/*c*	Mo *K*α radiation, λ = 0.71073 Å
*a* = 11.7655 (9) Å	Cell parameters from 6618 reflections
*b* = 20.4640 (17) Å	θ = 2.0–25.9°
*c* = 6.7332 (5) Å	µ = 3.04 mm^−^^1^
β = 90.161 (9)°	*T* = 173 K
*V* = 1621.1 (2) Å^3^	Rod, colourless
*Z* = 4	0.30 × 0.11 × 0.10 mm

### 5-Bromo-6,8-dimethoxy-3-methyl-1H-isochromen-1-one chloroform monosolvate (II) Data collection

**Table:**

STOE IPDS 1 diffractometer	3131 independent reflections
Radiation source: fine-focus sealed tube	2066 reflections with *I* > 2σ(*I*)
Plane graphite monochromator	*R*_int_ = 0.087
φ rotation scans	θ_max_ = 25.9°, θ_min_ = 2.0°
Absorption correction: multi-scan (MULABS; Spek, 2020)	*h* = −14→14
*T*_min_ = 0.894, *T*_max_ = 1.000	*k* = −25→25
3131 measured reflections	*l* = 0→8

### 5-Bromo-6,8-dimethoxy-3-methyl-1H-isochromen-1-one chloroform monosolvate (II) Refinement

**Table:**

Refinement on *F*^2^	Primary atom site location: structure-invariant direct methods
Least-squares matrix: full	Secondary atom site location: difference Fourier map
*R*[*F*^2^ > 2σ(*F*^2^)] = 0.040	Hydrogen site location: mixed
*wR*(*F*^2^) = 0.100	H-atom parameters constrained
*S* = 0.88	*w* = 1/[σ^2^(*F*_o_^2^) + (0.0526*P*)^2^] where *P* = (*F*_o_^2^ + 2*F*_c_^2^)/3
3131 reflections	(Δ/σ)_max_ < 0.001
211 parameters	Δρ_max_ = 0.57 e Å^−^^3^
0 restraints	Δρ_min_ = −0.40 e Å^−^^3^

### 5-Bromo-6,8-dimethoxy-3-methyl-1H-isochromen-1-one chloroform monosolvate (II) Special details

**Table:**

Geometry. All esds (except the esd in the dihedral angle between two l.s. planes) are estimated using the full covariance matrix. The cell esds are taken into account individually in the estimation of esds in distances, angles and torsion angles; correlations between esds in cell parameters are only used when they are defined by crystal symmetry. An approximate (isotropic) treatment of cell esds is used for estimating esds involving l.s. planes.
Refinement. Refined as a 2-component twin. BASF = 0.2590 (19)2-axis ( 0 0 1 ) [ 0 0 1 ], Angle () [] = 0.16 Deg, Freq = 49 ************* (-1.000 0.000 0.000) (h1) (h2) Nr Overlap = 3120 ( 0.000 -1.000 0.000) * (k1) = (k2) BASF = 0.15 ( 0.003 0.000 1.000) (l1) (l2) DEL-R =-0.011

### 5-Bromo-6,8-dimethoxy-3-methyl-1H-isochromen-1-one chloroform monosolvate (II) Fractional atomic coordinates and isotropic or equivalent isotropic displacement parameters (Å^2^)

**Table:**

	*x*	*y*	*z*	*U*_iso_*/*U*_eq_	Occ. (<1)
Br1	0.17186 (4)	0.18589 (2)	0.76040 (12)	0.03702 (17)	
O1	0.6171 (3)	0.37438 (16)	0.7786 (8)	0.0409 (11)	
O2	0.4383 (2)	0.40139 (13)	0.7725 (6)	0.0265 (8)	
O3	0.3644 (3)	0.09462 (14)	0.7647 (7)	0.0386 (9)	
O4	0.6719 (3)	0.24940 (16)	0.7829 (7)	0.0390 (9)	
C1	0.3284 (4)	0.2074 (2)	0.7669 (9)	0.0245 (11)	
C2	0.4075 (4)	0.1564 (2)	0.7681 (9)	0.0281 (11)	
C3	0.5225 (4)	0.1697 (2)	0.7730 (9)	0.0292 (12)	
H3	0.575634	0.134698	0.774737	0.035*	
C4	0.5612 (4)	0.2337 (2)	0.7756 (9)	0.0260 (12)	
C5	0.5216 (4)	0.3538 (2)	0.7769 (9)	0.0249 (11)	
C6	0.3248 (4)	0.3877 (2)	0.7700 (8)	0.0258 (11)	
C7	0.2865 (4)	0.32622 (19)	0.7680 (9)	0.0215 (10)	
H7	0.207010	0.318184	0.765122	0.026*	
C8	0.3647 (4)	0.2720 (2)	0.7702 (8)	0.0204 (10)	
C9	0.4828 (4)	0.2862 (2)	0.7722 (8)	0.0203 (10)	
C10	0.4443 (5)	0.0418 (2)	0.7595 (12)	0.0515 (15)	
H10A	0.486906	0.040490	0.884718	0.077*	
H10B	0.497084	0.048281	0.648942	0.077*	
H10C	0.403488	0.000461	0.741327	0.077*	
C11	0.7509 (5)	0.1961 (3)	0.7800 (16)	0.069 (2)	
H11A	0.742784	0.171933	0.655046	0.104*	
H11B	0.735424	0.166759	0.891818	0.104*	
H11C	0.828565	0.213036	0.791178	0.104*	
C12	0.2546 (4)	0.4485 (2)	0.7733 (11)	0.0395 (14)	
H12A	0.283364	0.479241	0.673878	0.047*	
H12B	0.259135	0.468449	0.905318	0.047*	
H12C	0.175335	0.437600	0.742878	0.047*	
C20	0.8802 (5)	0.3955 (4)	0.7872 (12)	0.062 (2)	
H20	0.799342	0.381292	0.801403	0.075*	
Cl1	0.93131 (17)	0.41367 (10)	1.0251 (3)	0.0703 (6)	
Cl2A	0.8846 (9)	0.4482 (4)	0.6107 (16)	0.130 (4)	0.5
Cl3A	0.9643 (6)	0.3226 (3)	0.7218 (19)	0.100 (3)	0.5
Cl2B	0.8631 (8)	0.4812 (4)	0.6905 (16)	0.104 (3)	0.5
Cl3B	0.9684 (6)	0.3556 (4)	0.6389 (15)	0.134 (4)	0.5

### 5-Bromo-6,8-dimethoxy-3-methyl-1H-isochromen-1-one chloroform monosolvate (II) Atomic displacement parameters (Å^2^)

**Table:**

	*U*^11^	*U*^22^	*U*^33^	*U*^12^	*U*^13^	*U*^23^
Br1	0.0368 (3)	0.0338 (2)	0.0405 (3)	−0.0140 (2)	0.0035 (3)	−0.0009 (4)
O1	0.0160 (16)	0.0347 (19)	0.072 (3)	−0.0049 (14)	0.001 (2)	0.003 (2)
O2	0.0222 (16)	0.0183 (14)	0.039 (2)	0.0000 (12)	0.0005 (19)	0.0003 (19)
O3	0.066 (2)	0.0165 (16)	0.034 (2)	−0.0002 (14)	0.002 (2)	−0.003 (2)
O4	0.0244 (17)	0.0432 (19)	0.049 (3)	0.0139 (16)	−0.003 (2)	0.001 (2)
C1	0.028 (2)	0.022 (2)	0.023 (3)	−0.0028 (18)	0.006 (3)	−0.006 (2)
C2	0.045 (3)	0.021 (2)	0.018 (3)	−0.001 (2)	0.004 (3)	0.000 (3)
C3	0.044 (3)	0.025 (2)	0.019 (3)	0.017 (2)	0.001 (3)	0.005 (2)
C4	0.024 (2)	0.032 (2)	0.022 (3)	0.0108 (19)	0.002 (2)	0.000 (3)
C5	0.026 (3)	0.026 (2)	0.022 (3)	−0.0002 (19)	−0.002 (2)	0.000 (3)
C6	0.026 (2)	0.025 (2)	0.027 (3)	0.0016 (19)	0.000 (3)	0.002 (3)
C7	0.0186 (19)	0.026 (2)	0.020 (3)	0.0001 (16)	−0.002 (2)	−0.002 (3)
C8	0.025 (2)	0.023 (2)	0.014 (3)	−0.0008 (17)	0.000 (2)	−0.005 (3)
C9	0.022 (2)	0.023 (2)	0.016 (3)	−0.0009 (17)	−0.001 (2)	0.001 (2)
C10	0.091 (4)	0.022 (2)	0.041 (4)	0.018 (3)	0.000 (5)	0.004 (3)
C11	0.033 (3)	0.063 (4)	0.112 (7)	0.027 (3)	−0.004 (5)	−0.006 (5)
C12	0.029 (2)	0.027 (3)	0.063 (4)	0.006 (2)	0.000 (3)	0.003 (3)
C20	0.025 (3)	0.092 (5)	0.070 (6)	−0.002 (3)	0.001 (3)	0.009 (5)
Cl1	0.0589 (11)	0.0721 (12)	0.0799 (15)	−0.0165 (9)	−0.0007 (10)	0.0057 (11)
Cl2A	0.129 (7)	0.121 (7)	0.138 (9)	−0.055 (6)	−0.047 (6)	0.096 (6)
Cl3A	0.028 (2)	0.074 (3)	0.198 (9)	−0.002 (2)	0.013 (4)	−0.042 (5)
Cl2B	0.094 (4)	0.100 (5)	0.119 (8)	−0.026 (4)	−0.037 (4)	0.055 (5)
Cl3B	0.035 (3)	0.204 (9)	0.164 (9)	−0.011 (6)	0.007 (4)	−0.136 (8)

### 5-Bromo-6,8-dimethoxy-3-methyl-1H-isochromen-1-one chloroform monosolvate (II) Geometric parameters (Å, º)

**Table:**

Br1—C1	1.894 (4)	C7—H7	0.9500
O1—C5	1.201 (5)	C8—C9	1.419 (6)
O2—C6	1.364 (5)	C10—H10A	0.9800
O2—C5	1.382 (5)	C10—H10B	0.9800
O3—C2	1.361 (5)	C10—H10C	0.9800
O3—C10	1.433 (6)	C11—H11A	0.9800
O4—C4	1.343 (6)	C11—H11B	0.9800
O4—C11	1.434 (6)	C11—H11C	0.9800
C1—C8	1.389 (6)	C12—H12A	0.9800
C1—C2	1.398 (6)	C12—H12B	0.9800
C2—C3	1.381 (7)	C12—H12C	0.9799
C3—C4	1.387 (6)	C20—Cl2A	1.605 (12)
C3—H3	0.9500	C20—Cl3B	1.657 (10)
C4—C9	1.416 (6)	C20—Cl1	1.749 (8)
C5—C9	1.457 (6)	C20—Cl3A	1.844 (10)
C6—C7	1.335 (6)	C20—Cl2B	1.882 (11)
C6—C12	1.494 (6)	C20—H20	1.0000
C7—C8	1.441 (6)		
			
C6—O2—C5	123.3 (3)	C8—C9—C5	120.1 (4)
C2—O3—C10	117.2 (4)	O3—C10—H10A	109.5
C4—O4—C11	116.5 (4)	O3—C10—H10B	109.5
C8—C1—C2	120.4 (4)	H10A—C10—H10B	109.5
C8—C1—Br1	121.3 (3)	O3—C10—H10C	109.5
C2—C1—Br1	118.3 (3)	H10A—C10—H10C	109.5
O3—C2—C3	123.2 (4)	H10B—C10—H10C	109.5
O3—C2—C1	116.5 (4)	O4—C11—H11A	109.5
C3—C2—C1	120.3 (4)	O4—C11—H11B	109.5
C2—C3—C4	120.5 (4)	H11A—C11—H11B	109.5
C2—C3—H3	119.7	O4—C11—H11C	109.5
C4—C3—H3	119.7	H11A—C11—H11C	109.5
O4—C4—C3	123.0 (4)	H11B—C11—H11C	109.5
O4—C4—C9	116.8 (4)	C6—C12—H12A	109.5
C3—C4—C9	120.2 (4)	C6—C12—H12B	109.4
O1—C5—O2	114.6 (4)	H12A—C12—H12B	109.5
O1—C5—C9	128.8 (4)	C6—C12—H12C	109.5
O2—C5—C9	116.5 (4)	H12A—C12—H12C	109.5
C7—C6—O2	121.6 (4)	H12B—C12—H12C	109.5
C7—C6—C12	126.7 (4)	Cl2A—C20—Cl1	121.6 (6)
O2—C6—C12	111.7 (4)	Cl3B—C20—Cl1	116.2 (5)
C6—C7—C8	120.6 (4)	Cl2A—C20—Cl3A	110.4 (7)
C6—C7—H7	119.7	Cl1—C20—Cl3A	102.0 (5)
C8—C7—H7	119.7	Cl3B—C20—Cl2B	108.5 (6)
C1—C8—C9	119.7 (4)	Cl1—C20—Cl2B	98.9 (5)
C1—C8—C7	122.5 (4)	Cl2A—C20—H20	107.4
C9—C8—C7	117.8 (4)	Cl1—C20—H20	107.4
C4—C9—C8	118.8 (4)	Cl3A—C20—H20	107.4
C4—C9—C5	121.0 (4)		
			
C10—O3—C2—C3	−2.1 (9)	C2—C1—C8—C9	0.8 (8)
C10—O3—C2—C1	178.1 (6)	Br1—C1—C8—C9	−179.1 (4)
C8—C1—C2—O3	180.0 (5)	C2—C1—C8—C7	179.6 (6)
Br1—C1—C2—O3	−0.1 (7)	Br1—C1—C8—C7	−0.2 (8)
C8—C1—C2—C3	0.2 (9)	C6—C7—C8—C1	−179.6 (6)
Br1—C1—C2—C3	−179.9 (5)	C6—C7—C8—C9	−0.7 (8)
O3—C2—C3—C4	179.8 (6)	O4—C4—C9—C8	−178.2 (5)
C1—C2—C3—C4	−0.4 (9)	C3—C4—C9—C8	1.2 (8)
C11—O4—C4—C3	2.5 (9)	O4—C4—C9—C5	−0.1 (8)
C11—O4—C4—C9	−178.1 (6)	C3—C4—C9—C5	179.4 (6)
C2—C3—C4—O4	179.1 (6)	C1—C8—C9—C4	−1.4 (8)
C2—C3—C4—C9	−0.3 (9)	C7—C8—C9—C4	179.6 (5)
C6—O2—C5—O1	180.0 (5)	C1—C8—C9—C5	−179.6 (5)
C6—O2—C5—C9	1.9 (8)	C7—C8—C9—C5	1.4 (8)
C5—O2—C6—C7	−1.2 (9)	O1—C5—C9—C4	2.1 (10)
C5—O2—C6—C12	177.8 (5)	O2—C5—C9—C4	179.8 (5)
O2—C6—C7—C8	0.5 (9)	O1—C5—C9—C8	−179.8 (6)
C12—C6—C7—C8	−178.3 (6)	O2—C5—C9—C8	−2.0 (8)

### 5-Bromo-6,8-dimethoxy-3-methyl-1H-isochromen-1-one chloroform monosolvate (II) Hydrogen-bond geometry (Å, º)

**Table:**

*D*—H···*A*	*D*—H	H···*A*	*D*···*A*	*D*—H···*A*
C10—H10*C*···O1^i^	0.98	2.59	3.511 (6)	156
C11—H11*C*···Cl3*A*	0.98	2.79	3.629 (9)	144
C20—H20···O1	1.00	2.15	3.126 (6)	164

Symmetry code: (i) −*x*+1, *y*−1/2, −*z*+3/2.
